# Understanding Barriers Impacting upon Patient Wellbeing: A Nationwide Italian Survey and Expert Opinion of Dermatologists Treating Patients with Moderate-to-Severe Psoriasis

**DOI:** 10.3390/jcm13010101

**Published:** 2023-12-24

**Authors:** Francesca Prignano, Giuseppe Argenziano, Federico Bardazzi, Riccardo G. Borroni, Alexandra M. G. Brunasso, Martina Burlando, Anna Elisabetta Cagni, Elena Campione, Elisa Cinotti, Fabrizio Colonna, Aldo Cuccia, Stefano Dastoli, Rocco De Pasquale, Clara De Simone, Vito Di Lernia, Valentina Dini, Gabriella Fabbrocini, Claudia Galluzzi, Alfredo Giacchetti, Claudia Giofrè, Claudia Lasagni, Serena Lembo, Francesco Loconsole, Maria Antonia Montesu, Paolo Pella, Stefano Piaserico, Paolo Pigatto, Antonio Giovanni Richetta, Adriana Scuotto, Elena Stroppiana, Marina Venturini, Anna Stefania Vinci, Leonardo Zichichi, Maria Concetta Fargnoli

**Affiliations:** 1 Department of Health Sciences, Section of Dermatology, University of Florence, 50125 Florence, Italy; 2 Dermatology Unit, University of Campania, 80138 Naples, Italy; g.argenziano@gmail.com; 3 Dermatology Unit, IRCCS Azienda Ospedaliero-Universitaria di Bologna, Policlinico S. Orsola-Malpighi, 40138 Bologna, Italy; federico.bardazzi@aosp.bo.it; 4 Department of Medical and Surgical Sciences, Alma Mater Studiorum University of Bologna, 40126 Bologna, Italy; 5 Department of Biomedical Sciences, Humanitas University, 20089 Milan, Italy; riccardoborroni@gmail.com; 6 Dermatology Unit, Humanitas Research Hospital, IRCCS, 20089 Milan, Italy; 7 Department of Dermatology, Villa Scassi Hospital, ASL 3, 16149 Genova, Italy; giovanna.brunasso@gmail.com; 8 Dermatologic Clinic, DISSAL, San Martino Policlinico San Martino Hospital, 16132 Genova, Italy; martinaburlando@hotmail.com; 9 Unità Operativa Dipartimentale di Dermatologia e Venereologia, IRCCS San Gerardo, 20900 Milan, Italy; annaelisabetta.cagni@irccs-sangerardo.it; 10 Dermatologic Unit, Department of Systems Medicine, University of Rome Tor Vergata, 00133 Rome, Italy; campioneelena@hotmail.com (E.C.); claudia.galluzzi@gmail.com (C.G.); 11 Dermatology Unit, Department of Medical, Surgical and Neurological Sciences, University of Siena, 53100 Siena, Italy; elisacinotti@gmail.com; 12 Dipartimento di Psicologia, Università di Torino, 10124 Turin, Italy; fabrizio-colonna@virgilio.it; 13 Unit of Dermatology, San Donato Hospital, 52100 Arezzo, Italy; dott.aldocuccia@gmail.com; 14 Department of Health Sciences, Magna Graecia University of Catanzaro, 88100 Catanzaro, Italy; stefanodastoli@tiscali.it; 15 Dermatology Unit, San Marco Hospital, 95123 Catania, Italy; r.depasquale@unict.it; 16 Institute of Dermatology, Catholic University, 00185 Rome, Italy; clara.desimone@unicatt.it; 17 Dermatology Unit, Fondazione Policlinico Universitario A. Gemelli IRCCS, 00168 Rome, Italy; 18 Dermatology Unit, Arcispedale S. Maria Nuova, Azienda USL-IRCCS di Reggio Emilia, 42122 Reggio Emilia, Italy; dilernia.vito@ausl.re.it; 19 Unit of Dermatology, University of Pisa, 56126 Pisa, Italy; valentinadini74@gmail.com; 20 Section of Dermatology, Department of Clinical, Medicine and Surgery, University of Naples Federico II, 80138 Naples, Italy; 21 UOC Dermatology, IRCCS INRCA, 60124 Ancona, Italy; alfredogiacchetti@gmail.com; 22 Dermatology Complex Operative Unit, Papardo Hospital, 98158 Messina, Italy; claudiagiofre@tiscali.it; 23 AOU Policlinico di Modena, Department of Specialized Medicine, University of Modena, 41121 Modena, Italy; lasacla65@gmail.com; 24 Department of Medicine, Surgery and Dentistry, “Scuola Medica Salernitana”, University of Salerno, 84084 Fisciano, Italy; slembo@unisa.it; 25 Clinica Dermatologica, Azienda Ospedaliero Universitaria Consorziale Policlinico di Bari, 70124 Bari, Italy; franciscus59@gmail.com; 26 Department of Surgical, Microsurgical and Medical Sciences, Dermatology, University of Sassari, 07100 Sassari, Italy; mmontesu@uniss.it; 27 Dermatologia, Ospedale degli Infermi, 13875 Biella, Italy; paolo.pella@aslbi.piemonte.it; 28 Dermatology Unit, Department of Medicine, University of Padova, 35122 Padova, Italy; stefano.piaserico@gmail.com; 29 Clinical Dermatology, Department of Biomedical, Surgical and Dental Sciences, Istituto Ortopedico Galezzi, University of Milan, 20122 Milan, Italy; paolo.pigatto@unimi.it; 30 Unit of Dermatology, Department of Internal Medicine and Medical Specialties, Sapienza University of Rome, 00185 Rome, Italy; antonio.richetta@uniroma1.it; 31 Department of Advanced Biomedical Science, Legal Medicine Section, University of Naples Federico II, 80131 Naples, Italy; adriana.scuotto2@unina.it; 32 Section of Dermatology, Department of Medical Sciences, University of Turin, 10124 Turin, Italy; elena.stroppiana@gmail.com; 33 Dermatology Department, University of Brescia, ASST Spedali Civili, 25121 Brescia, Italy; marina.venturini@unibs.it; 34 Private Clinic, Via Cenisio 18, 20154 Milan, Italy; stefania.vinci1807@gmail.com; 35 Unit of Dermatology, San Antonio Abate Hospital, 80057 Trapani, Italy; dermatologia@asptrapani.it; 36 Dermatology, Department of Biotechnological and Applied Clinical Sciences, University of L’Aquila, 67100 L’Aquila, Italy; mariaconcetta.fargnoli@univaq.it

**Keywords:** psoriasis, dermatologist perspective, quality of life, treatment, wellbeing, patients, surveys and questionnaires

## Abstract

A nationwide cross-sectional online survey was administered to dermatologists managing patients with moderate-to-severe plaque psoriasis across Italy to obtain real-world dermatologists’ perspectives on the impact of psoriasis and its treatment on patients’ daily lives and quality of life (QoL). A total of 91 dermatologists (aged 39.1 ± 11.2 years) completed a 31-question survey and workshop sessions were undertaken in order to identify the best management approach to achieve patient wellbeing. Social (4.2 ± 0.1), physical (4.26 ± 0.2) and mental components (4.1 ± 0.3) were rated by dermatologists as contributing to patient wellbeing to similar extents. While a high proportion (85.4%; rating of 4.3 out of 5) of dermatologists felt that they considered the QoL of patients, a lower proportion (69.6%; rating of 3.7 out of 5) felt that patients were satisfied in this regard. The psoriasis area and severity index and body surface area were the instruments most frequently used to assess the physical domain, while interviews/questions and the dermatology life quality index were used to assess social and mental domains, with only 60% of dermatologists following up on these aspects. The importance of investigating the presence of comorbidities was recognized but not always carried out by many dermatologists, (>70%), particularly for obesity and anxiety/depression. This survey identified key components contributing to barriers impacting on the QoL of patients with moderate-to-severe psoriasis from the perspective of the dermatologist.

## 1. Introduction

Plaque psoriasis is a chronic inflammatory skin disease that affects approximately 2–3% of patients worldwide [1]. As well as physical disability, patients experience a profound impact on their psychological wellbeing, resulting in an impaired quality of life (QoL) [2,3].

While the psoriasis area and severity index (PASI), body surface area (BSA) or physician global assessment (PGA) [4,5] are used to assess the severity of the disease, the QoL of patients with psoriasis is mainly assessed by the dermatology life quality index (DLQI) or the short-form (SF-36) health survey [6].

Biological therapies, such as tumor necrosis factor (TNF), interleukin (IL)-17 and IL-23 inhibitors and small molecules allow dermatologists to successfully treat moderate-to-severe psoriasis [7,8,9] and improve patients’ QoL [10,11,12]. Despite this, many patients remain untreated/undertreated, decline or fail to respond or experience side effects [13].

Psoriasis can significantly impact a patient’s self-image, leading to embarrassment due to visible lesions, resulting in low self-esteem, anxiety and depression, suicide attempts and suicide [14,15,16]. A recent study performed in Italy, including 208 patients with plaque psoriasis, showed that the prevalence of depressive symptoms was 14.9% and that of suicide risk was 6.3% [17].

In this regard, the impact of psoriasis goes beyond the severity of skin lesions, as demonstrated by the lack of agreement between the QoL scores (e.g., DLQI) and clinical severity (i.e., PASI) [18,19]. As many as half of patients feel that their dermatologists do not fully understand the impact of the disease on their mental health [20]. In this regard, the perception of QoL is now considered a critical measure in this setting [21,22] and is recognized by dermatologists as an urgent unmet need, as also suggested by European guidelines for the treatment of psoriasis [23].

We have previously collected information from patients to understand the impact of psoriasis on their wellbeing and QoL, using an online survey. This survey found that only 23.8% of patients believed their dermatologist took their wellbeing into account, and 32.6% of patients considered their therapy inadequate to improve the signs and symptoms of the disease [24]. The present paper’s aim was to use an online survey to collect information on the wellbeing and management of psoriasis patients from the perspective of the dermatologist in order to identify key barriers to be overcome to improve these patients’ QoL.

## 2. Materials and Methods

### 2.1. Project Design

The “SHAPE” (SHAring Patient Experiences) study was a prospective cross-sectional nationwide survey undertaken in adult patients with plaque psoriasis involving dermatologists from 32 centers across the four main macro areas of Italy (North-West, North-East, Center and South). Using an online 26-question survey, the first part of the SHAPE study evaluated the impact of psoriasis on patients’ wellbeing from the perspective of the patient; the methodology and results have been described in detail elsewhere [24].

**Figure 1:**
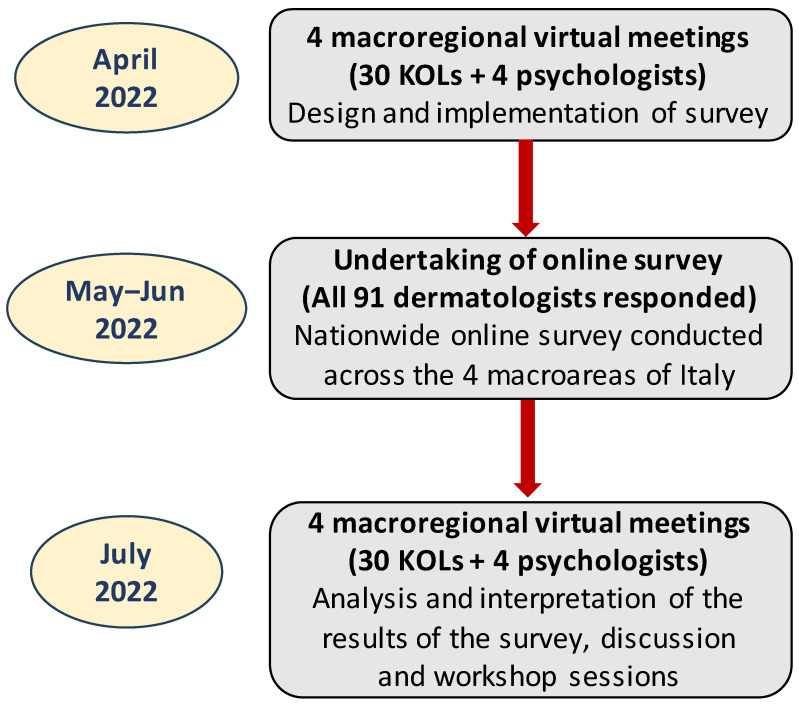
Flow chart of different stages undertaken before and during implementation of the online patient survey.

Using a modified version of this online survey, the present nationwide survey aimed to gather information from the perspective of the dermatologist to identify key barriers preventing the improvement of patients’ QoL and wellbeing. This survey was conducted in three phases: (a) four web-based meetings in April 2022 (one for each macro area) were undertaken between four psychologists and 30 key opinion leaders (KOLs), each representing one of the 30 centers, to discuss the design and implementation of the survey; (b) a total of 91 dermatologists with extensive experience in the management of patients with psoriasis completed the online survey; (c) four independent macroregional virtual meetings were held between the 30 KOLs and the four psychologists (who did not answer the survey) to discuss and interpret results from the survey (Figure 1).

### 2.2. Online Survey

In the present survey, dermatologists were prospectively asked to complete the survey through a dedicated website (http://www.surveymonkey.com; accessed on 10 June 2023) and included 31 specific questions relating to each dermatologist’s socio-demographic information and clinical experience (Q:1–10) and to patients’ wellbeing and management of psoriasis (Q:11–31) that could be completed in about 10 min (Supplementary Materials S1). Questions relating to patients’ wellbeing and management of psoriasis were designed by dermatologists and psychologists and aimed to collect information (from the perspective of the dermatologist) on the therapeutic/management approach to the patient in real-life clinical practice, focusing on the psychological/emotional component, doctor–patient communication, therapeutic adherence and availability (during visits).

The original survey was in Italian (Supplementary Materials S1) and a translated version in English is available (Supplementary Materials S2).

### 2.3. Statistical Analysis

Data are presented using mean ± SD or number and %. Scores for some variables are presented as box-whisker plots showing median and interquartile range. Comparisons between patients’ and dermatologists’ scores for physical, social and emotional wellbeing domains were performed by the Students *t* test. Comparisons between the number (frequency) of dermatologists for specific measures/assessments were performed by the Chi-squared test. Data derived from the online survey are summarized as number and %. A *p*-value of ≤0.05 was considered statistically significant and analysis was performed using MedCalc software (version 12.2.1.0., Mariakerke, Belgium).

## 3. Results

### 3.1. Characteristics of Dermatologists

The online survey was completed by 91 dermatologists, evenly distributed throughout Italy (Table 1). Question 1–10 of the survey were related to general demographics and experience with psoriasis patients; this information is summarized in Table 1. The mean age of participants was 39.1 ± 11.2 years and the majority (75.8%) were qualified dermatologists based in a hospital or university clinic, of which approximately one-third (31.4%) had patients with psoriasis, 67.9% of whom had moderate-to-severe psoriasis.

### 3.2. Perspective from Dermatologists on Physical, Social and Psychological Domains

**Figure 2:**
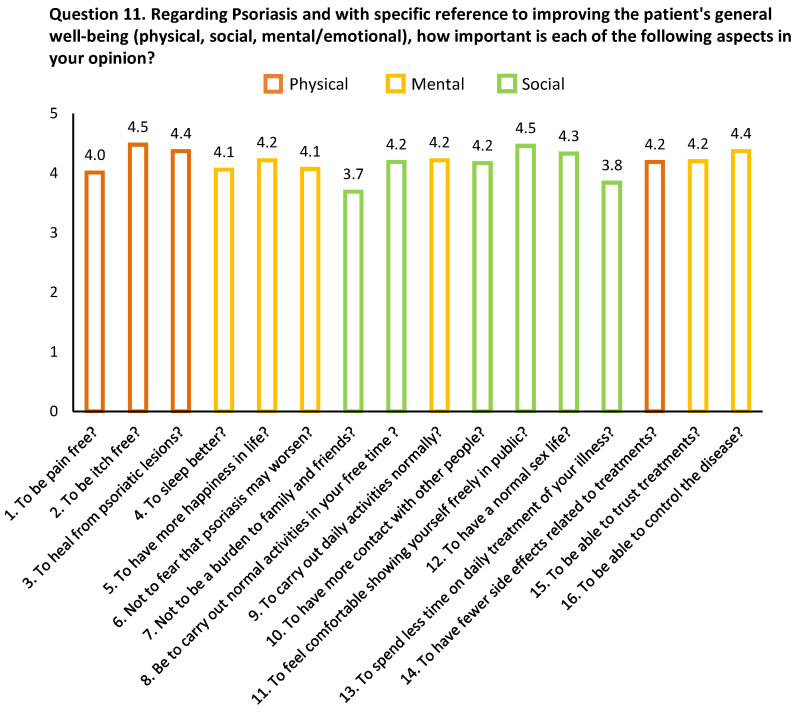
Rating of three core domains, physical, mental and social, associated with achievement of patient wellbeing. Results from questions/statements related to Question 11 from the online questionnaire. (Question 11. Regarding Psoriasis and with specific reference to improving the patient’s general well-being (physical, social, mental/emotional), how important is each of the following aspects in your opinion?) Data are presented as mean ± SD.

Dermatologists’ perspectives on physical, social and psychological domains were assessed through Question 11 in the online questionnaire. Using a scale from 0–5 (0 representing the lowest relevance and 5 the most important), questions/issues relating to the three core domains relative to patient wellbeing (i.e., physical, mental and social) were rated. Sixteen questions related to the three domains revealed similar scores ranging from 3.69 (“Not to be a burden to family and friends”) to 4.46 (“To feel comfortable showing yourself freely in public”) (Figure 2). Dermatologists gave the three domains similar importance, although the physical component was found to be the highest (4.26 ± 0.2), followed by social (4.2 ± 0.1) and mental (4.1 ± 0.3). We also used multivariate analysis to explore predictor variables that could be potentially associated with Question 11 scores. Female gender was found to be significantly associated with higher scores (*β* = 0.23, *p* = 0.026), independent of age, percentage of patients with psoriasis, years treating patients with psoriasis or number of patients treated per year (Table 2). The overall mean score for female dermatologists was significantly higher than for males (4.3 ± 0.4 vs. 4.0 ± 0.5, *p* = 0.002). Furthermore, of all 16 items relating to Question 11, the average score was higher for females, attaining statistical significance for eight items (Supplementary Materials S3).

### 3.3. Dermatologists’ Perspective on Patient QoL

Question 12 and 13 of the survey focused on patients’ QoL and their satisfaction of the way in which the dermatologist takes into consideration aspects relating to QoL. The overall rating of the dermatologists was 4.25 ± 0.7, with 76 (85.4%) dermatologists feeling that they considered QoL aspects of psoriasis patients “very much” or “a lot”, while the rating was lower (3.7 ± 0.9), and when asked how satisfied they thought patients were with their dermatologist’s consideration of QoL aspects, 59.6% said “very much” or “a lot” (Table 3).

### 3.4. Dermatologists’ Perspectives on Practice Conditions, Patient Communication and Their Satisfaction

Dermatologists responded to specific questions (Question 14–21) related to visit conditions and general communication with patients during consultation (Table 4). Only 30–40% of dermatologists regarded practice conditions during consultation as optimal (Question 14 and 16), in the relatively short time (mean 18.8 ± 5.8 min; range 10–45 min) for each visit (Question 15). Interestingly, when stratified by gender, female dermatologists reported having a significantly longer mean visit duration than male dermatologists (19.9 ± 6.3 min vs. 17.4 ± 4.9 min, *p* = 0.04). Although the majority of dermatologists asked patients, “How are you?” or “How are you feeling?” during visits (89.9%), only half of dermatologists kept track of the answer in subsequent visits (Question 17 and 18). While the importance of dialogue was considered important in evaluating the type of patient during the visit by 76.4% of dermatologists (Question 19; rating of 4.1 ± 0.5), a dialogue to observe aspects/issues (not measurable by other means, such as instruments or scales) of the patient during the visit was not considered very important by about 30% of dermatologists (mean rating score of 3.85 ± 0.4; Question 20), while a high proportion of dermatologists considered it important to investigate disease history (83.2%; Question 21 of the survey).

### 3.5. Use of Surveys and Scales to Assess Patients with Psoriasis

**Figure 3:**
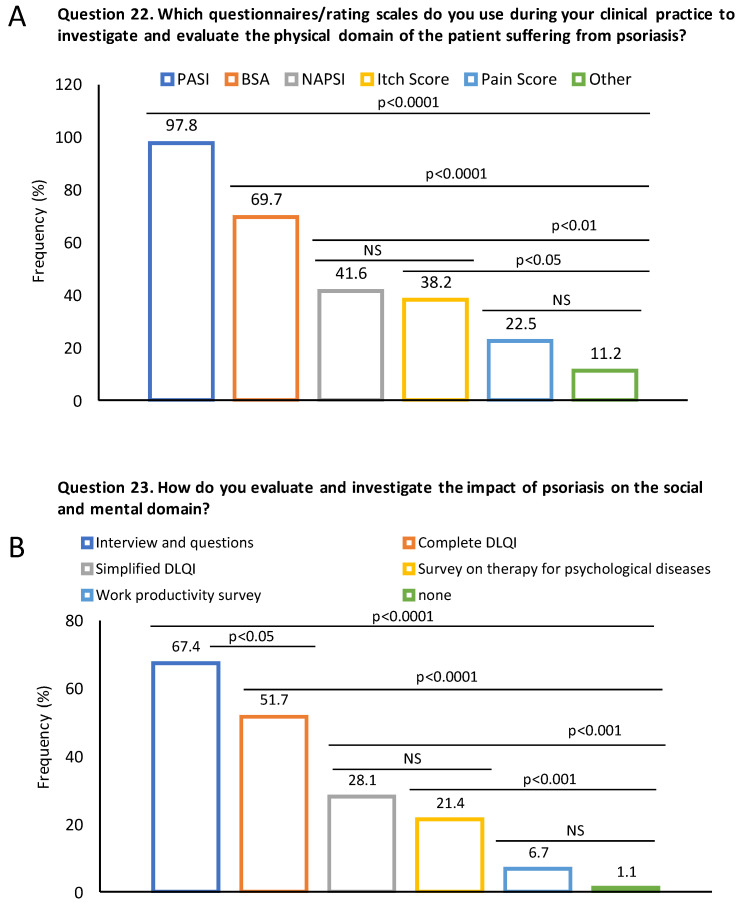
Instruments/scales used by dermatologists to assess physical and mental domains in patients with psoriasis. (**A**) Proportion of dermatologists using different questionnaires/scales to evaluate physical domain according to Question 22 from the questionnaire (“Which questionnaires/rating scales do you use during your clinical practice to investigate and evaluate the physical domain of the patient suffering from psoriasis?”). (**B**) Proportion of dermatologists using different questionnaires/scales to evaluate physical domain according to Question 23 from the questionnaire (“How do you evaluate and investigate the impact of psoriasis on the social and mental domain?”). Levels of statistical significance between the frequency of instruments/scales are shown.

The scores most frequently used to evaluate the physical domain were the psoriasis area severity index (PASI; 97.8%) and/or body surface area (BSA; 69.7%; Figure 3A), while interviews/questions (67.4%) and the dermatology life quality index (DLQI; simple or complete; ~80%) were used to assess mental/social domains (Question 22 and 23 of the survey) (Figure 3B).

### 3.6. Dermatologists’ Perspective on Evaluation of Comorbidities and Monitoring of Lesions in Difficult-to-Treat Areas

While a high proportion (>90%) of dermatologists stated that they sometimes or always monitored the progress of physical and mental/social aspects of patients (Question 24), only 26.9% and 7.9%, always investigated cases of excess body weight or anxiety/depression, respectively (Question 25–26 and Question 28; Table 5).

**Figure 4:**
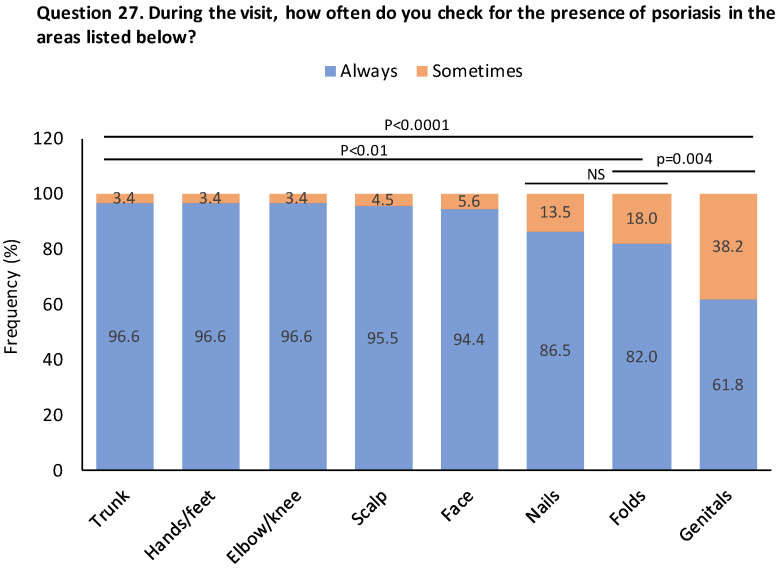
The proportion of dermatologists who always or sometimes checked for the presence of psoriasis in specific areas. Data are presented as %. Levels of statistical significance between the frequency of specific lesions sometimes checked by dermatologists are shown.

While ~95% of dermatologists stated that they always examined the trunk, hands/feet, elbows/knees, scalp and face during consultation, nails (13.5%), folds (18%) and genital areas (38.2%) were only “sometimes” examined (Figure 4).

### 3.7. Discussion with Patient on Previous or Current Treatment

Dermatologists answered specific questions related to the request for information about previous and current treatments (Question 29–31; Table 6). Although a high proportion (92.1%) of dermatologists always verified that patients understood the treatment that they were receiving (Question 29), 76% felt that they had the opportunity in their routine practice to discuss with patients’ family members or carers how they could support the patient (Question 30). In addition, only 43.8% of dermatologists always investigated the reason/motivation why the patient contacted them after being previously treated by another dermatologist.

### 3.8. Output from Interactive Workshop Session

Taking into consideration the results derived from the online patients’ survey, combined with dermatologists’ experience and the current literature, interactive web-based workshop sessions were undertaken to explore specific areas in detail.

The key summary points discussed during these workshop sessions are summarized below.

#### 3.8.1. Importance of Awareness of the Social and Mental Domain and Communication between Patient and Physician

Between 33% and 46% of dermatology patients reported clinically significant symptoms of anxiety [25,26,27] and worries associated with social anxiety [28]. It has also been estimated that between 25% and 40% of dermatology patients have a psychological disorder that is underreported [29].

Dermatologists cannot underestimate the importance of doctor–patient communication and the importance of using simple language. Words should be clear, sentences short and calmly spoken. The climate should be welcoming, enabling the person to see that their feelings of pain, anger, shame and helplessness are recognized and to perceive a real empathetic attitude. It is important for the dermatologist to help the person balance the positive and negative aspects of the disease and reduce the sense of helplessness.

Determined both by the presence of visible lesions and by people’s lack of knowledge of the disease, as well as linked to social and cultural factors [30], discrimination and humiliation are very commonly experienced by patients (84%) [31]. Discrimination has impacts on work, intimacy and general health. Patients describe daily psychological difficulties associated with the exposure of their body (e.g., in the gym, in the swimming pool, at the hairdresser or at work).

It is important to note that as many as 27% of the population are unwilling to have a relationship with someone who has psoriasis [32]. In individuals with psoriasis, emotional reactions frequently follow one another rapidly and range from alarm to fear, despondency, shame, sense of helplessness and loss of control. The dermatologist can represent a “reservoir” or “empty space” to receive all these emotions. What can be returned is a better knowledge of the disease, greater attention to the most obvious signs of suffering and a continuous sharing of therapeutic objectives. In more difficult cases, it is appropriate for the dermatologist to consult a specialist or ask for a consultation.

#### 3.8.2. Need for Therapeutic Approach to Achieve Patient Wellbeing

Following discussion among dermatologists regarding treatment options with the aim of achieving patient wellbeing, several points were agreed upon.

In multi-failure patients with a long history of disease and different risk factors/underlying conditions, long-term efficacy and adherence to therapy are important. In these cases, an anti-IL-23 agent may be a good therapeutic choice.

Regarding anti-TNF-α (originators or biosimilars), there are patients who are completely satisfied with the treatment, such as “complete responders” and patients who do not respond or lose response to anti-TNF-α or who have comorbidities where anti-TNF-α biologics are contraindicated; in these cases, anti-IL-23 or anti-IL-17 are appropriate. Notably, anti-TNF-α biologics may be associated with side-effects, which must be communicated to the patient.

In real-life practice, anti-IL-17 biologics are prescribed when there is a need for a quick response. On the other hand, IL-23 biologics can offer additional benefits over other biologics in a range of patient types. A summary of these benefits is listed in Table 7.

Dermatologists also emphasized that placing the patient’s QoL at the center (main focus) can lead to a more targeted choice of treatments. With a view to calibrating the therapeutic choice, novel, simple and standardized questionnaires could help identify the best drug for the patient (“tailored approach”) based on their needs and QoL, as well as considerably shortening visit duration. Focus on instruments/tools was discussed separately in detail and summarized below.

#### 3.8.3. Instruments/Tools Available in Real-Life Practice to Assess Patient Wellbeing

In real-life clinical practice, the duration of visits was reported to be under 20 min on average, leaving dermatologists limited time to complete questionnaires or tools to evaluate patient QoL.

While almost all dermatologists used the PASI or BSA to assess the physical domain, the DLQI was used to assess the social/mental domain by about 80% of dermatologists, as well as dialogue. The DLQI is a simple questionnaire consisting of 10 questions that can be used for any skin disease in routine clinical practice [33]. However, previous studies observed that clinical severity measurements almost invariably showed a poor correlation with different indexes of QoL and psychological distress in patients with psoriasis [34]. The dissimilarity between clinical severity assessment and patient-centered measures stresses the need for a more comprehensive assessment of severity of psoriasis.

However, mean PASI and DLQI correlate predictably in patients with moderate-to-severe psoriasis undergoing treatment with biologics [35] and achievement of PASI75/90/100 can translate to significant QoL improvement in patients treated with biologics. In the DERMBIO registry, correlation between changes in PASI and DLQI in 1677 patients with moderate-to-severe psoriasis treated with biologics or apremilast for five years showed a weak-to-moderate correlation between PASI and DLQI [36]. In a separate study undertaken in Germany, patients who declared a DLQI item to be relevant showed a higher disease severity and a lower health state [37]. The items most frequently marked as ‘not relevant’ were item 7 (“work and study”, 28.1%), item 6 (“sport, exercise”, 26.0%), item 9 (“sexual relationships”, 22.1%) and item 8 (“personal relationships”, 15.8%). Considering an item to be “not relevant” because of disease-related disabilities to participate in sports, social events and other activities of everyday life (high disease burden) should be treated differently in HRQoL assessment undertaken by means of the DLQI. The DLQI-Relevant (DLQI-R) is a recently developed scoring that adjusts the total score of the questionnaire for the number of not relevant responses indicated by a patient [38].

In patients with morphea, pemphigus and psoriasis, DLQI-R scoring improves the discriminatory power of the questionnaire by benefiting from the additional information on non-relevant responses. However, it is also recognized that changing the scoring may undermine the greatest strengths of the DLQI: the vast quantity of data in the current literature based on the DLQI and its original scoring scheme, comparison of the relative impact of various diseases and treatments with well-established standards and attempting to improve upon an already excellent tool might come at the expense of making the tool much less valuable [39].

The hospital anxiety and depression scale (HADS) is used to assess anxiety in patients in a range of settings and HADS has been used to evaluate the effect of different biologics in patients with moderate-to-severe psoriasis, with improvement in anxiety and depressive symptoms being observed [40]. However about 80% of dermatologists stated that they never used an instrument/scale to assess anxiety or depression in psoriasis patients in clinical practice. Understanding the disease and treatment experience of patients and their opinions using validated satisfaction tools and psychometric attitudes scales is crucial to improving the doctor–patient relationship and the active role of patients in treatment decision making.

## 4. Discussion

This cross-sectional nationwide survey was completed by 91 dermatologists with the aim of evaluating the dermatologists’ perspectives on the impact of psoriasis and its treatment on patients’ daily lives and QoL. In our first SHAPE survey, we evaluated the impact of psoriasis using a 26-question survey that was completed by patients with moderate-to-severe psoriasis [24]. We identified key factors contributing to barriers impacting upon patient wellbeing. In the present 31-question survey, we identified several areas to improve patient care and management.

While we observed that dermatologists rated social, physical and mental components as contributing to a similar extent, as far as importance for patient wellbeing is concerned, female dermatologists (on average) gave greater importance (mean scores were significantly higher) for all 16 issues relating to the three domains, as well as a slightly (but significantly) longer time during visits (approximately 2 min longer on average, compared to males). A recent observational study conducted in the US including 102,664 visits with 405 physicians revealed that visits with female physicians were significantly longer than those with male physicians [41]. Furthermore, patient satisfaction was higher for female physicians vs. males, but this higher level of satisfaction was not associated with increased visit duration. It is also recognized that patient expectation by physician gender can vary [42]. One study found female physicians displayed more patient-centeredness, but this did not strongly correlate with patient satisfaction [43]. For male physicians who practiced patient-centered communication, this was associated with higher satisfaction, suggesting gendered expectations for physician behavior and communication [43]. Another study observed that patient satisfaction was associated to different gender attributes. Patients tended to like female physicians, whose style of communication was in line with their gender role (e.g., leaning towards the patient and using a soft voice) [44]. Interestingly, in our first patient-based survey, no difference was observed between male and female patients with regards to their scores for QoL or wellbeing [24]. While we cannot explain why scores were higher for female dermatologists than for males, nor the longer duration of visits, these phenomena may be linked to potential expectation from the patient. Whether these gender-specific differences may be associated with differences in patient satisfaction remains to be established in this setting.

With regard to the importance of patient wellbeing (Question 11 in the survey, which was identical to that in our previous study, completed by patients [24]), we observed that dermatologists rated 12/16 (75%) of the statements as having less importance (mean score of 4.18 ± 0.21) compared to patients (mean score 4.36 ± 0.18, *p* = 0.0004) (Supplementary Materials S4). Three of the four statements rated by dermatologists as more important than the patients did were based on mental domains (Statements 10–12; “To be able to have more contact with other people”, “To feel comfortable showing yourself freely in public?” and “To be able to have a normal sex life?” while one statement was rated as similar among dermatologists and patients (Statement no. 2 “To be itch free?” rated as 4.48 and 4.46 by dermatologists and patients, respectively). While approximately 40% of patients reported that the dermatologist did not take (little or nothing) their state of well-being into consideration, about 24% of patients reported that the dermatologist was interested in their state of well-being. These values contrast markedly with those for dermatologists, who completed these same questions.

While a high proportion (85.4%; rating of 4.3) of dermatologists felt that they considered the QoL of patients, a lower proportion of dermatologists (69.6%; rating of 3.7) felt that patients were satisfied in this regard. Approximately one-third of the dermatologists acknowledged that the conditions under which the examination took place were inadequate for optimal results. Although dermatologists recognized the importance of communication with the patient, this was not always undertaken. PASI and BSA were the instruments most frequently used to assess the physical domain, while interviews/questions and DLQI were used to assess social and mental domains, with only 60% of dermatologists following up on these aspects. Dermatologists recognized the importance of investigating the presence of concomitant diseases but in a high proportion (>70%) this was not always investigated, particularly excess body weight and anxiety/depression. Considering that as many as one-third of patients with moderate-to-severe psoriasis are obese [45], an awareness of the need to investigate this comorbidity further and referral to a specialist that can offer advice and management would be warranted.

It is also recognized that the social and psychological impact caused by psoriasis is generally underestimated by dermatologists [30,31,32] and as many as half of patients feel that their healthcare professionals do not understand the mental health impact of the disease [20]. In our first patient-based survey, we observed that about 40% of patients felt that their dermatologist was not taking their wellbeing into consideration [24]. In contrast, a high proportion of dermatologists (85.4%) in the present survey (Question 12 and 13), felt that they considered the QoL of patients, pointing towards a clear gap between the perspectives of patient and physician. The ability to communicate empathetically with patients has been shown to have a positive effect in clinical practice, in addition to establishing a trusting relationship [46]. A study performed in Italy supports this view, showing that the dermatologist’s interpersonal skills are the most important factor to have a likely positive effect on treatment adherence and health outcomes and therefore improve patient satisfaction [47].

## 5. Study Limitations

The number of dermatologists who participated in this survey (N = 91) was low, but they were distributed across the Italian territory and can therefore be considered representative. The online survey was based on 31 questions to cover issues and areas that could impact on patients’ QoL and wellbeing. However, the questionnaire was not validated, although it was based on three validated questionnaires (PBI, DLQI and the WHO-5 wellbeing index). It was also designed by four experts with extensive experience in the management of patients with psoriasis and a particular interest in the wellbeing and QoL of patients with this disease. For some of the questions in the survey, two dermatologists skipped/did not answer the questions. The importance of smoking and psoriasis is recognized [48,49] and this component was not explored from the perspective of the dermatologist in this survey.

## 6. Conclusions

In this survey, dermatologists answered 31 questions relating to the wellbeing and management of patients with psoriasis.

Psoriatic patients should be assessed from a holistic point of view. This concept is multidimensional, encompassing the physical, social and psychological wellbeing of the individual and based on the patient’s view of their condition.

Apart from the clinical severity of affected areas, psoriasis can also have a profound psychological impact on the patient’s QoL. It is becoming important that dermatologists quantify the impact of psoriasis on patients’ lives and the ways in which therapy improves QoL.

It is also important to document if and how the patient’s lifestyle is affected by psoriasis, what the patient perceives as the most bothersome aspects of their psoriasis and its treatment and, finally, what their hopes and expectations are from attendance at treatment clinics.

To address this issue, collaboration between dermatologists and psychologists is fundamental. In this respect, the development of an algorithm in collaboration with psychologists to define the best patient approach for dermatologists is warranted. This knowledge would simplify formulation of the treatment plan with more appropriate individual goals and may contribute to improved compliance with treatment advice. Future studies using novel tools, as well as specialized training for dermatologists to improve adherence in the use and implementation of tools and techniques to monitor and improve patient wellbeing are warranted.

## Figures and Tables

**Table 1:** Characteristics of dermatologists participating in the survey.

Characteristic	Dermatologists (N = 91)
Region of Italy of practice	
North-West	32 (35.2)
North-East	9 (9.8)
Center	26 (28.6)
South	24 (26.4)
Female gender, n (%)	50 (54.9)
Age (years)	39.1 ± 11.2
Profession/title	
Dermatologist	69 (75.8)
Resident (specialising in dermatology)	22 (24.2)
Main area where you see patients	
Hospital	52 (57.1)
University clinic	41 (45.1)
Private ambulatory	13 (14.3)
Specialist ambulatory	8 (8.8)
Experience managing psoriasis patients	
<1 year	3 (3.3)
≥1 year	88 (96.7)
Time (years)	10.6 ± 8.9
Number of patients treated annually (any dermatological illness)	1844 ± 1781 (10–8000)
Percentage of patients with psoriasis	31.4 ± 23.1
Disease severity according to PASI (%)	
Mild	32.1 ± 18.6
Moderate	38.8 ± 14.5
Severe	29.1 ± 17.7

PASI = psoriasis area severity index. Data are presented as mean ± standard deviation or number and %.

**Table 2:** Multivariate linear regression analysis of variables associated with mean score for 16 items from Question 11 of the survey.

Characteristic	*β*-Coefficient	Standard Error	*t*-Statistic	*p*-Value
Age, years	0.006	0.008	0.74	0.46
Gender (female/male)	0.23	0.1	2.3	**0.026**
Years treating psoriatic patients	0.006	0.01	0.63	0.53
Percentage psoriatic patients	0.004	0.002	1.66	0.1
Percentage with severe disease	0.00004	0.003	0.014	0.99
Number of patients visited/year	−0.00002	0.00003	−0.6	0.55

Question 11: Regarding Psoriasis and with specific reference to improving the patient’s general well-being (physical, social, mental/emotional), how important is each of the following aspects in your opinion? *p*-values in bold text denote statistically significant associations.

**Table 3:** Dermatologists’ perspectives on the QoL of patients.

Question and Rating	Dermatologists (N = 89) * N (%)	Mean Score (0–5)
Question 12. How much do you think you take into consideration aspects relating to the quality of life of your patients with psoriasis (work sphere, social relationships, psychological state)?		
Not at all	0 (0.0)	
A little	0 (0.0)	
Quite a lot	13 (14.6)	4.3 ± 0.7
Very much	41 (46.1)	
A lot	35 (39.3)	
Question 13. How satisfied do you think patients with psoriasis (in general) are with their dermatologist’s consideration of aspects relating to quality of life (work sphere, social relationships, psychological state)?		
Not at all	0 (0.0)	
A little	8 (8.99)	
Quite a lot	28 (31.5)	3.7 ± 0.9
Very much	36 (40.5)	
A lot	17 (19.1)	

* Two dermatologists skipped/did not respond to this question.

**Table 4:** Dermatologists’ perspectives on communication and other information during a visit.

Question and Rating	Dermatologists (N = 89) * N (%)	Mean Score (0–5)
Question 14. Do you think that in your clinical practice the conditions in which you visit patients with psoriasis are optimal for achieving a good therapeutic alliance?		
No	4 (4.9)	
Not always	33 (37.1)	NR
Yes	52 (58.4)	
Question 16. Do you have an adequate space/setting for the quiet and confidential conduct of the visit that can put the patient suffering from psoriasis at ease?		
No	4 (4.9)	
Not always	22 (24.7)	NR
Yes	63 (70.8)	
Question 17. During the visit, do you explicitly ask the patient suffering from psoriasis “how are you” or “how are you feeling”?		
No, never	0 (0.0)	
Yes, sometimes	9 (10.1)	NR
Yes, always	80 (89.9)	
Question 18. When you ask the patient “how are you” or “how are you feeling”, do you keep track of the answer and follow its progress during subsequent visits?		
No, never	8 (8.99)	
Yes, sometimes	35 (39.3)	NR
Yes, always	46 (51.7)	
Question 19. Regarding psoriasis, how important do you think it is to carry out a conversational survey to identify the type of patient during the visit?		
Not at all	0 (0.0)	
A little	1 (1.12)	
Quite a lot	20 (22.5)	4.1
Very much	37 (41.6)	
A lot	31 (34.8)	
Question 20. Regarding psoriasis, how important do you think it is to observe the non-quantifiable aspects of the patient (non-verbal communication, clothing, other) during the visit?		
Not at all	0 (0.0)	
A little	3 (3.4)	
Quite a lot	27 (30.3)	3.85
Very much	39 (43.8)	
A lot	20 (22.5)	
Question 21. Regarding psoriasis, how important do you think it is to investigate the patient’s disease history during the visit?		
Not at all	0 (0.0)	
A little	0 (0.0)	
Quite a lot	15 (16.9)	4.16
Very much	45 (50.6)	
A lot	29 (32.6)	

* Two dermatologists skipped/did not respond to this question. NR = not relevant.

**Table 5:** Questions relating to patient follow up and presence of other concomitant diseases.

Question and Rating	Dermatologists (N = 89) * N (%)
Question 24. With reference to question 23, do you keep track of these aspects and follow their progress?	
No, never	5 (5.6)
Yes, sometimes	31 (34.8)
Yes, always	53 (59.6)
Question 25. Do you carry out investigations in case of excess weight of the patient suffering from psoriasis?	
No, never	9 (10.1)
Yes, sometimes	56 (62.9)
Yes, always	24 (26.9)
Question 26. Do you investigate the presence of joint pain in patients suffering from psoriasis?	
No, never	0 (0.0)
Yes, sometimes	9 (10.1)
Yes, always	80 (89.9)
Question 28. Do you use questionnaires/rating scales to investigate the presence of anxiety and depression in patients suffering from psoriasis?	
No, never	69 (77.5)
Yes, sometimes	13 (14.6)
Yes, always	7 (7.9)

* Two dermatologists skipped/did not respond to this question.

**Table 6:** Questions relating to discussion of previous and current treatment.

Question and Rating	Dermatologists (N = 89) * N (%)
Question 29. In your clinical practice, do you usually verify the patient’s understanding of the therapy and clinical indications for the treatment of psoriasis?	
No, never	1 (1.1)
Yes, sometimes	6 (6.7)
Yes, always	82 (92.1)
Question 30. In your clinical practice, where necessary and if the patient suffering from psoriasis allows it, do you have the opportunity to discuss with family members or caregivers to make them understand how they can be of support?	
No, never	2 (2.7)
Yes, sometimes	18 (21.3)
Yes, always	69 (76.0)
Question 31. In the case of a patient previously treated by another dermatologist, during the visit do you investigate the motivation that led him to contact you?	
No, never	14 (15.7)
Yes, sometimes	36 (40.45)
Yes, always	39 (43.8)

* Two dermatologists skipped/did not respond to this question.

**Table 7:** Summary of main recommendations/expert opinion for the use of IL-17 or IL-23 to achieve wellbeing and some advantages of IL-23 biologics over other biologics.

Use of Anti-IL-23 by Patient Type	Recommendation/Expert Opinion
Chronic inflammatory intestinal disease	Anti-IL-17 not recommended
Obesity	IL-23 recommended
Recurrent infection	Anti-IL-17 not recommended for Candida infection
Recurrent respiratory disease	Only limited use of IL-17
Liver disease	Often infectious disease specialist directs towards safer drugs
Atopic dermatitis	Potential worsening or onset of dermatitis when using anti-IL-17
Benefits of anti-IL-23 use:	
With anti-IL-23 biologics the patient is able to obtain a clinical response. This type of response can also be extended to patients with inflammatory bowel diseases and other comorbidities. The suppressive effect on memory T cells potentially modifies the disease. The advantage conferred by more spaced administration times leads to a positive impact on the patient’s quality of life and to longer follow-up visits for patients with a predictably good response. There is safety for infectious comorbidities: during a period of increased risk of the spread of viral diseases, safety becomes an important issue. The response is stable over long periods of time, even if they are interrupted (compared to maintenance times of other therapies). Stability in responder maintenance is conferred. This innovative drug confers an expectation of achieving remarkable goals.	

## Data Availability

Data can be made available from the corresponding author upon request.

## Supplementary Materials

The following supporting information can be downloaded at: https://www.mdpi.com/article/10.3390/jcm13010101/s1, Supplementary Materials S1: Italian version of Online survey (Survey Monkey) used to evaluate obtain real-world dermatologists’ perspectives on the impact of psoriasis and its treatment on patients’ daily lives and quality of life; Supplementary Materials S2: English version of Online survey (Survey Monkey) used to evaluate obtain real-world dermatologists’ perspectives on the impact of psoriasis and its treatment on patients’ daily lives and quality of life; Supplementary Materials S3: Differences between male and female dermatologists’ with regard to their perspectives on physical, social and psychological domains as assessed through Question 11 of the online questionnaire; Supplementary Materials S4: Radar plot depicting rating of 16 different statements specific to Question 11 from the survey related to physical, social and emotional wellbeing from the perspective of dermatologists and patients.
