# Supplementary material for: Understanding Barriers Impacting upon Patient Wellbeing: A Nationwide Italian Survey and Expert Opinion of Dermatologists Treating Patients with Moderate-to-Severe Psoriasis
Source: J Clin Med. 2023 Dec 24;13(1):101. doi: 10.3390/jcm13010101 (PMC10779771; doi:10.3390/jcm13010101)
Supplement: Supplementary file 1 [file jcm-13-00101-s001.zip › Supplementary Materials S1.pdf]

Supplementary Table S1

Versione Italiana

<u>**Informazioni demografiche generali ed esperienza nel campo della psoriasi**</u>

1. **Regione di provenienza del compilatore?**
2. **Età del compilatore?**
3. **Sesso?**
4. **Professione e disciplina?**
5. **In che ambito prevalente svolge la Sua attività clinica?** (possibilità di selezionare più di una risposta)
6. **Da quanto tempo si occupa di Psoriasi nella Sua pratica clinica?**
7. **Indichi da quanti anni si occupa di Psoriasi nella Sua pratica clinica**
8. **Quanti pazienti affetti da patologie dermatologiche tratta annualmente?** (Si prega di fornire il numero in riferimento all'ultimo anno solare)
9. **Quale è la percentuale dei pazienti affetti da Psoriasi sul totale complessivo dei Suoi pazienti?** (Si prega di fornire una stima in percentuale, sul totale dei Suoi pazienti, riferita all'ultimo anno solare)
10. **Come si distribuiscono i Suoi pazienti con Psoriasi, in percentuale, in base alla classificazione PASI?** (Si prega di fornire una stima in percentuale, sul totale dei Suoi pazienti con Psoriasi, riferita all'ultimo anno solare. La somma delle risposte deve risultare = 100%)

<u>**La perspettiva del dermatologo sul benessere fisico, sociale ed emotivo della psoriasi**</u>

11. **Riguardo alla Psoriasi e con riferimento specifico al miglioramento del benessere generale del paziente (fisico, sociale, mentale/emotivo), quanto è importante secondo il Suo giudizio ognuno dei seguenti aspetti?** (scegli da; per niente, poco, abbastanza, molto, moltissimo)

1. essere libero dal dolore
2. essere libero dal prurito
3. guarire dalle lesioni psoriasiche
4. dormire meglio
5. avere più gioia di vivere
6. potersi sentire libero dalla paura che la psoriasi possa peggiorare
7. non essere un peso per parenti e amici
8. poter svolgere delle attività normali nel tempo libero (attività con amici, con familiari, sportive)
9. essere in grado di svolgere normalmente le attività quotidiane (es. fare la spesa/occuparsi della casa o del giardino oppure lavorare oppure studiare)
10. poter avere più contatti con altra gente

11. sentirsi a proprio agio nel mostrarsi liberamente in pubblico
12. poter avere una vita sessuale normale
13. poter dedicare meno tempo alla cura quotidiana della sua malattia
14. aver meno effetti collaterali legati ai trattamenti
15. poter avere fiducia nei trattamenti
16. poter controllare la malattia

12. **Quanto pensa di tenere in considerazione gli aspetti relativi alla Qualità di Vita dei Suoi pazienti con Psoriasi** (sfera lavorativa, relazioni sociali, stato psicologico)?
13. **Quanto pensa che i pazienti con Psoriasi (in generale) si ritengano soddisfatti della considerazione del proprio dermatologo circa gli aspetti relativi alla Qualità di Vita** (sfera lavorativa, relazioni sociali, stato psicologico)?
14. **Ritiene che nella Sua pratica clinica le condizioni in cui svolge la visita dei pazienti con Psoriasi siano ottimali per raggiungere una buona alleanza terapeutica** (condivisione di obiettivi tra paziente e specialista)?
15. **Quanto tempo (minuti) riesce a dedicare mediamente alla visita con il paziente affetto da Psoriasi?**
16. **Dispone di uno spazio/setting adeguato per lo svolgimento tranquillo e riservato della visita che possa mettere a proprio agio il paziente affetto da Psoriasi?**
17. **Durante la visita chiede esplicitamente al paziente affetto da Psoriasi "come sta" o "come si sente"?**
18. **Quando chiede al paziente "come sta" o "come si sente", tiene traccia della risposta e ne segue l'andamento durante le visite successive?**
19. **Riguardo alla Psoriasi, quanto ritiene importante effettuare un'indagine colloquiale per inquadrare la tipologia di paziente durante la visita?**
20. **Riguardo alla Psoriasi, quanto ritiene importante osservare gli aspetti non quantificabili del paziente (comunicazione non verbale, abbigliamento, altro) durante la visita?**
21. **Riguardo alla Psoriasi, quanto ritiene importante effettuare un'indagine della storia di malattia del paziente durante la visita?**
22. **Quali questionari/scale di valutazione utilizza durante la Sua pratica clinica per indagare e valutare il dominio fisico nel paziente affetto da Psoriasi?**
23. **Come valuta e indaga l'impatto della Psoriasi sul dominio sociale e mentale?(possibilità di selezionare più di una risposta)**
24. **In riferimento alla domanda N.23, tiene traccia di questi aspetti e ne segue l'andamento?**
25. **Effettua indagini in caso di eccesso ponderale del paziente affetto da Psoriasi?**
26. **Effettua indagini sulla presenza di dolori articolari nel paziente affetto da Psoriasi?**

27. **Durante la visita con quale frequenza controlla la presenza di Psoriasi a livello delle aree sotto elencate?**
28. **Utilizza questionari/scale di valutazione per indagare la presenza di ansia e depressione nei pazienti affetti da Psoriasi?**
29. **Nella Sua pratica clinica è solito verificare la comprensione del paziente rispetto alla terapia e alle indicazioni cliniche per la cura della Psoriasi?**
30. **Nella Sua pratica clinica, laddove necessario e se il paziente affetto da Psoriasi lo consente, ha modo di confrontarsi con i familiari o caregivers per far loro comprendere come poter essere di supporto?**
31. **In caso di paziente precedentemente in cura presso altro dermatologo, durante la visita indaga sulla motivazione che lo ha portato a rivolgersi a Lei?**
