# Supplementary material for: Understanding Barriers Impacting upon Patient Wellbeing: A Nationwide Italian Survey and Expert Opinion of Dermatologists Treating Patients with Moderate-to-Severe Psoriasis
Source: J Clin Med. 2023 Dec 24;13(1):101. doi: 10.3390/jcm13010101 (PMC10779771; doi:10.3390/jcm13010101)
Supplement: Supplementary file 1 [file jcm-13-00101-s001.zip › Supplementary Materials S2.pdf]

Supplementary Table S2

Online survey available from Survey Monkey website https://www.surveymonkey.com/

English version

General demographic information and experience in the area of psoriasis

1. **What region do your practice from in Italy?**
2. **What is your age?**
3. **What is your gender?**
4. **What is your profession/title?**
5. **What is the main area that you perform your clinical activity?** (possibility to select more than one answer)
6. **How long have you been managing patients with psoriasis in your clinical practice?** (less or more than one year)
7. **Please indicate how many years you have been managing patients with psoriasis in your clinical practice**
8. **How many patients suffering from dermatological diseases do you treat annually?** (please provide the number for the latest calendar year)
9. **What percentage of patients suffering from psoriasis out of the total number of your patients?** (please provide an estimate as a percentage of your total patients for the last calendar year)
10. **How are your patients with psoriasis distributed, in percentage, based on the PASI classification?** (Please provide an estimate as a percentage of the total number of your patients with Psoriasis, referring to the last calendar year. The sum of the answers must be = 100%)

Dermatologist's perspective on psoriasis physical, social and emotional well-being

11. **Regarding Psoriasis and with specific reference to improving the patient's general well-being (physical, social, mental/emotional), how important is each of the following aspects in your opinion?** (choose from; not at all, a little, quite a lot, very much, a lot)

    1. To be pain free?
    2. To be itch free?
    3. To heal psoriatic lesions?
    4. To sleep better?
    5. To have more joy to live?
    6. To be able to feel free from the fear that psoriasis may get worse?
    7. Not to be a burden to family and friends?

8. To be able to carry out normal activities in your free time (activities with friends, family, or sports)?
9. To be able to carry out daily activities normally (shopping / looking after the house or garden or working or studying)?
10. To be able to have more contact with other people?
11. To feel comfortable showing yourself freely in public?
12. To be able to have a normal sex life?
13. To spend less time on daily treatment of your illness?
14. To have fewer side effects related to treatments?
15. To be able to trust treatments?
16. To be able to control the disease?

12. **How much do you think you take into consideration aspects relating to the quality of life of your patients with psoriasis** (work sphere, social relationships, psychological state)?
13. **How satisfied do you think patients with Psoriasis (in general) are with their dermatologist's consideration of aspects relating to quality of life** (work sphere, social relationships, psychological state)?
14. **Do you think that in your clinical practice the conditions in which you visit patients with psoriasis are optimal for achieving a good therapeutic alliance** (sharing of objectives between patient and specialist)?
15. **How much time (minutes) can you dedicate on average to the visit with the patient suffering from psoriasis?**
16. **Do you have an adequate space/setting for the quiet and confidential conduct of the visit that can put the patient suffering from psoriasis at ease?**
17. **During the visit, do you explicitly ask the patient suffering from psoriasis "how are you" or "how are you feeling"?**
18. **When you ask the patient "how are you" or "how are you feeling", do you keep track of the answer and follow its progress during subsequent visits?**
19. **Regarding psoriasis, how important do you think it is to carry out a conversational survey to identify the type of patient during the visit?**
20. **Regarding psoriasis, how important do you think it is to observe the non-quantifiable aspects of the patient** (non-verbal communication, clothing, other) **during the visit?**
21. **Regarding psoriasis, how important do you think it is to investigate the patient's disease history during the visit?**
22. **Which questionnaires/rating scales do you use during your clinical practice to investigate and evaluate the physical domain of the patient suffering from psoriasis?** (possibility to select more than one answer from the following: PASI, BSA itch score, pain score, NAPSI, none, other; specify)
23. **How do you evaluate and investigate the impact of psoriasis on the social and mental domain?** (possibility to select more than one answer from the following: complete DLQI, simplified DLQI, simple interview questions,

survey on current therapies for the treatment of psychological diseases, work productivity survey, none, other; specify)

24. **With reference to question no. 23, do you keep track of these aspects and follow their progress?**
25. **Do you carry out investigations in case of excess weight of the patient suffering from psoriasis?**
26. **Do you investigate the presence of joint pain in patients suffering from psoriasis?**
27. **During the visit, how often do you check for the presence of psoriasis in the areas listed below?** (please choose from the following: trunk, face, scalp, hands/feet genitals, elbows/knees, nails, folds)
28. **Do you use questionnaires/rating scales to investigate the presence of anxiety and depression in patients suffering from psoriasis?**
29. **In your clinical practice, do you usually verify the patient's understanding of the therapy and clinical indications for the treatment of psoriasis?**
30. **In your clinical practice, where necessary and if the patient suffering from psoriasis allows it, do you have the opportunity to discuss with family members or caregivers to make them understand how they can be of support?**
31. **In the case of a patient previously treated by another dermatologist, during the visit do you investigate the motivation that led him to contact you?**
