# Supplementary material for: Understanding Barriers Impacting upon Patient Wellbeing: A Nationwide Italian Survey and Expert Opinion of Dermatologists Treating Patients with Moderate-to-Severe Psoriasis
Source: J Clin Med. 2023 Dec 24;13(1):101. doi: 10.3390/jcm13010101 (PMC10779771; doi:10.3390/jcm13010101)
Supplement: Supplementary file 1 [file jcm-13-00101-s001.zip › Supplementary Materials S3.pptx]

## Slide 1
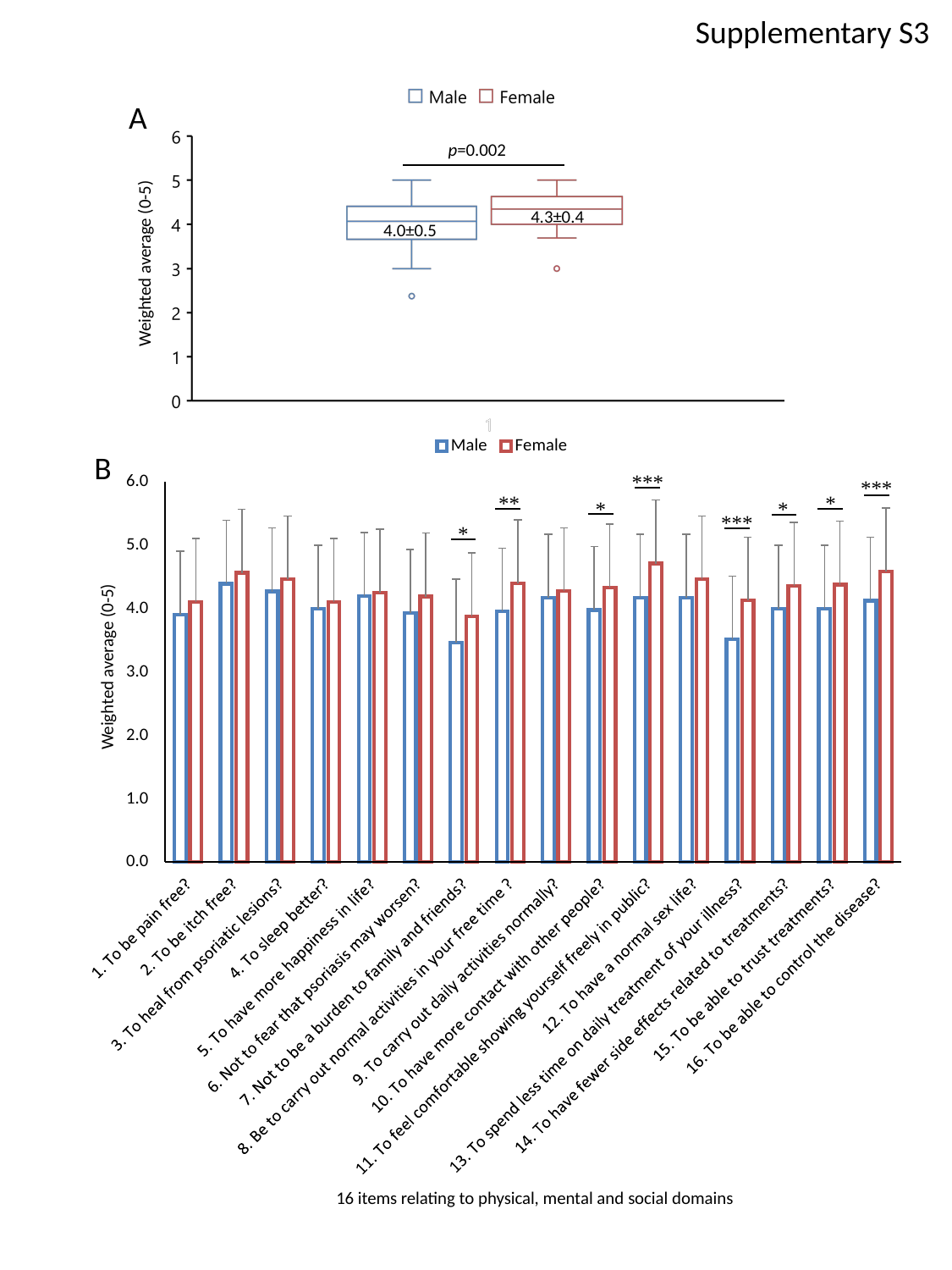

Supplementary S3
A
p=0.002
4.3±0.4
4.0±0.5
Weighted average (0-5)
B
***
***
*
**
*
*
***
*
Weighted average (0-5)
16 items relating to physical, mental and social domains
