# Supplementary material for: Understanding Barriers Impacting upon Patient Wellbeing: A Nationwide Italian Survey and Expert Opinion of Dermatologists Treating Patients with Moderate-to-Severe Psoriasis
Source: J Clin Med. 2023 Dec 24;13(1):101. doi: 10.3390/jcm13010101 (PMC10779771; doi:10.3390/jcm13010101)
Supplement: Supplementary file 1 [file jcm-13-00101-s001.zip › Supplementary Materials S4.pptx]

## Slide 1
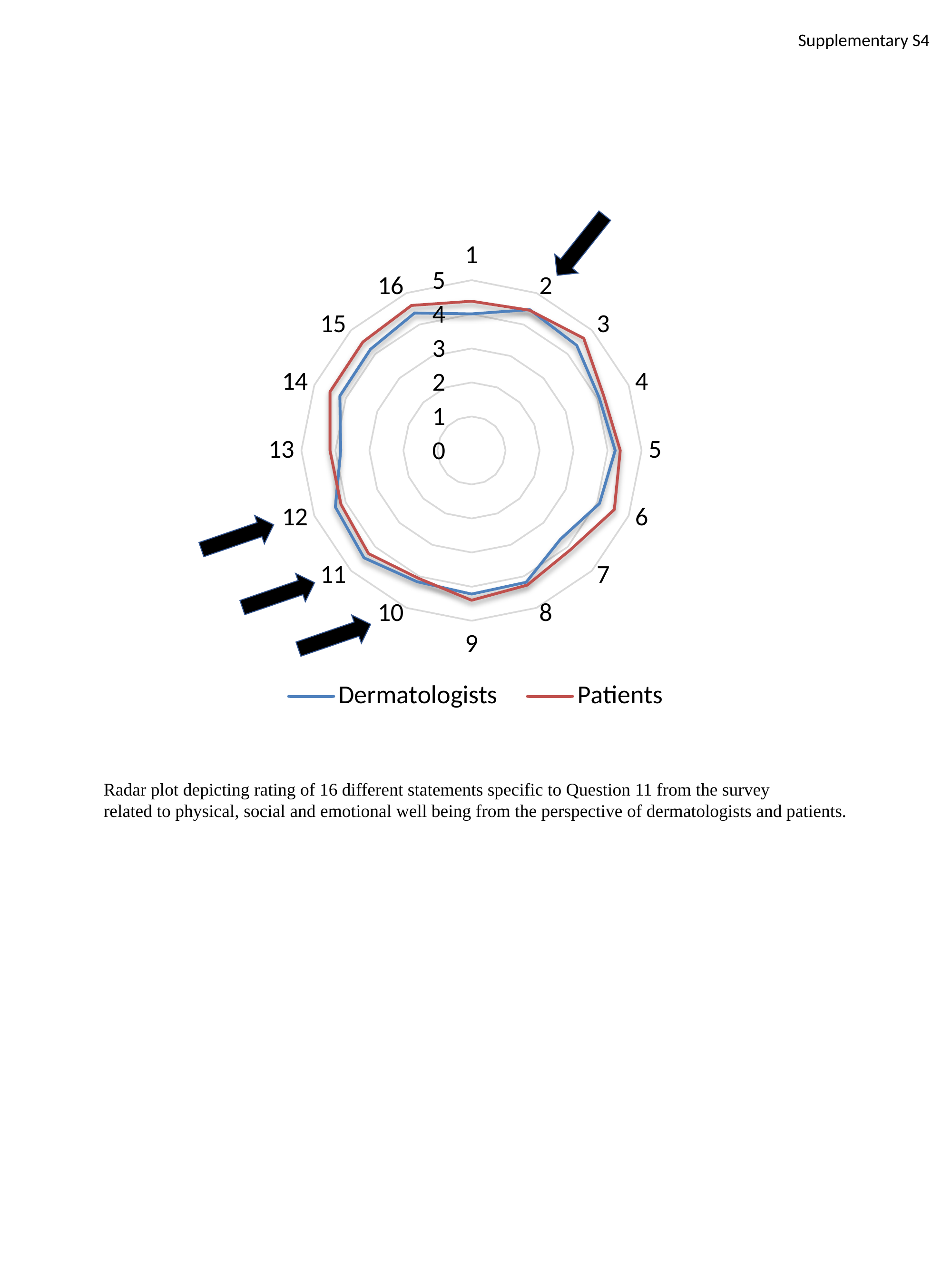

Supplementary S4
Radar plot depicting rating of 16 different statements specific to Question 11 from the survey
related to physical, social and emotional well being from the perspective of dermatologists and patients.
